# Development and Evaluation of a Web-Based Resource for Suicidal Thoughts: NowMattersNow.org

**DOI:** 10.2196/13183

**Published:** 2019-05-02

**Authors:** Ursula Whiteside, Julie Richards, David Huh, Rianna Hidalgo, Rebecca Nordhauser, Albert J Wong, Xiaoshan Zhang, David D Luxton, Michael Ellsworth, DeQuincy Lezine

**Affiliations:** 1 NowMattersNow.org Seattle, WA United States; 2 School of Social Work, University of Washington Seattle, WA United States; 3 Kaiser Permanente Washington Health Research Institute Seattle, WA United States; 4 Department of Health Services, University of Washington Seattle, WA United States; 5 Department of Psychiatry and Behavioral Sciences, University of Washington Seattle, WA United States; 6 Berkeley School of Law, University of California Berkeley, CA United States; 7 Civilization Seattle, WA United States; 8 Prevention Communities Fresno, CA United States

**Keywords:** dialectical behavior therapy, suicide, internet, help-seeking behavior, behavior therapy, crisis intervention, primary care, integrated health care systems

## Abstract

**Background:**

Nearly half of people who die by suicide see a health care provider in the month before their death. With the release of new care guidelines, detection of suicidal patients will likely increase. Providers need access to suicide-specific resources that can be used as part of immediate, brief interventions with a suicidal patient. Web-based suicide prevention resources have the potential to address this need.

**Objective:**

This study aimed to describe the development of the NowMattersNow.org website as a resource for individuals with suicidal thoughts and to evaluate the utility of the site via user experience surveys.

**Methods:**

NowMattersNow.org is an online video-based free public resource that provides evidence-based teachings, examples, and resources for managing suicidal thoughts and intense emotions focused largely around skills from dialectical behavior therapy. Developed with assistance from mental health consumers, it is intended to address gaps in access to services for suicidal patients in health care systems. Visitors stay an average of a minute and a half on the website. From March 2015 to December 2017, a user experience survey measured self-reported changes on a 1 (not at all) to 5 (completely overwhelming) scale regarding intensity of suicidal thoughts and negative emotions while on the website. Longitudinal regression analyses using generalized estimating equations evaluated the magnitude and statistical significance of user-reported changes in suicidal ideation and negative emotion. In secondary analyses, user-reported changes specific to subgroups, including men aged 36 to 64 years, mental health care providers, and other health care providers were evaluated.

**Results:**

During the period of analysis, there were 138,386 unique website visitors. We analyzed surveys (N=3670) collected during that time. Subsamples included men aged 36 to 64 years (n=512), mental health providers (n=460), and other health care providers (n=308). A total of 28% (1028/3670) of survey completers rated their suicidal thoughts as a 5 or “completely overwhelming” when they entered the website. We observed significant reductions in self-reported intensity of suicidal thoughts (–0.21, *P*<.001) and negative emotions (–0.32, *P*<.001), including decreases for users with the most severe suicidal thoughts (–6.4%, *P*<.001), most severe negative emotions (–10.9%, *P*<.001), and for middle-aged men (–0.13, *P*<001). Results remained significant after controlling for length of visit to website (before the survey) and technology type (mobile, desktop, and tablet).

**Conclusions:**

Survey respondents reported measurable reductions in intensity of suicidal thoughts and emotions, including those rating their suicidal thoughts as completely or almost completely overwhelming and among middle-aged men. Although results from this user-experience survey administered at one point in time to a convenience sample of users must be interpreted with caution, results provide preliminary support for the potential effectiveness of the NowMattersNow.org website as a tool for short-term management of suicidal thoughts and negative emotions.

## Introduction

### Background

Nearly half of people who die by suicide in the United States see some type of health care provider in the month before their death [1,2]. Newly released screening and care guidelines for suicidal patients [3] have the potential to increase the number of suicidal patients detected across health care settings. Unfortunately, most providers, particularly those in primary care settings where the majority of patients are seen before death by suicide, generally are not trained to work with suicidal patients [4,5]. There is also a lack of widely disseminated resources available for immediate support of suicidal patients, and the vast majority of people who die by suicide never receive specialized mental health care [6], with men being particularly unlikely to seek mental health care but far more likely to die by suicide than women [7,8].

Providers need access to suicide-specific resources that can be used as part of immediate, brief interventions with a suicidal patient. Web-based suicide-prevention resources are available around the clock and have the potential to address several major challenges to caring for suicidal patients [9,10]. Web-based services can assist providers as they are providing care by giving them a free resource to share, support suicidal patients during the time between identification and receipt of specialty mental health care, and serve as an adjunct intervention for suicidal patients who are not able to access specialty metal health care or prefer not to receive this type of care (eg, males). However, in reviewing the existing literature on Web-based prevention strategies for suicidal individuals, Jacob et al explicitly noted the lack of evidence-based resources and called for further development and evaluation of these tools [11]. A recent review of the small number of studies on the effectiveness of Web-based intervention websites (and mobile apps) reported some promise in terms of reductions in suicidal thoughts [12], although many of the interventions reviewed included barriers to access such as the need to set up an account or were difficult or impossible to access outside of the specific research trial.

Web-based resources that do not require the user to sign-in (ie, provide contact information or other details about their identity) can reduce important obstacles to use, including concerns about privacy. In the United States, examples of highly used, free Web-based resources include sites from the American Foundation for Suicide Prevention [13] and the National Suicide Prevention Lifeline [14]. Both of these sites provide important resources and self-help tips for suicide prevention but are not specifically tailored for a patient and clinician audience. The effectiveness of educational Web resources for suicide and self-directed violence prevention has historically been an area of research need [15]. Recent research indicates positive findings, for example, Till et al [16] reported results from a randomized trial that indicated that users with higher baseline scores of suicidality experienced an immediate reduction of suicidal thoughts compared with persons who visited a control website, thus indicating promise for the use of educative websites for brief intervention. Moreover, free Web-based resources may be particularly useful for middle-aged men in the United States who are unlikely to seek mental health care [17] and disproportionately impacted by suicide death [18].

### Objectives

NowMattersNow.org is a website that was developed based on the evidence provided above, for the purpose of supporting suicidal patients and providers who care for them ([19], see Multimedia Appendix 1). Central to the educative website are first-person stories of successful coping with and recovery from suicidal thoughts and painful emotions using strategies from a highly effective treatment for suicidal individuals (dialectical behavior therapy, DBT) [20-22]. Indeed, research indicates that video portrayals of recovery from suicide-related crisis may be beneficial to those experiencing higher rates of suicidal thoughts [23]. The purpose of this report was to describe the development and evaluation of this Web-based resource. For our evaluation, we present data from a brief user-experience survey collected from NowMattersNow.org users. The survey measures changes in suicidal thoughts and negative emotions while on the website and also collects demographic data on a high-risk group (men aged 36-64 years) and health care provider status. We hypothesized that survey completers, including those who rated their suicidal thoughts and negative emotions at the most intense end of the scale, would report reduced intensity after viewing the website. Secondary stratified analyses evaluated the impact on a smaller and higher risk group (middle age men) and providers (mental health and other health care providers) who may be using the website to help support suicidal patients rather than self-help. In addition, we investigated length of time to take the survey as an indicator of website “dose” as the length of exposure to the website may be associated with ratings of intensity of suicidal thoughts and negative emotions, and the impact of viewing the website on a mobile phone versus other device to inform our recommendations for website use.

## Methods

### NowMattersNow.org Origins

DBT is a robust evidence-based treatment that includes skills and self-management strategies (DBT skills) to change and tolerate emotions that drive suicidal thoughts [20-22,24]. However, frontline clinical providers such as those working in primary care have few resources to explain or connect patients to specialized mental health treatment, including DBT. Mental health consumer advocates have created websites to make DBT resources more widely available, for example, dbtselfhelp.com [25]. When brief DBT-based interventions began to demonstrate promise for treatment of suicidal patients [26-28], the first and second authors (motivated as researchers but also by personal and professional experiences–related suicidal patients, their providers, and perceived lack of resources) initiated research to begin the development of a DBT-inspired Web-based resource [19,29]. The results were used to design the NowMattersNow.org website. This was done in collaboration with Team Now Matters Now, a group of mental health consumers with suicidal experiences recruited for the project and the website design firm Civilization [30]. Most of the website content consists of videos of Team Now Matters Now members discussing their lived experience including using DBT skills to manage suicidal thoughts, thus providing social models of success [31]. The NowMattersNow.org website was launched on World Suicide Prevention Day in 2014 (September 10) and had more than 250,000 unique visitors as of December 2018.

### NowMattersNow.org Design

**Figure 1:**
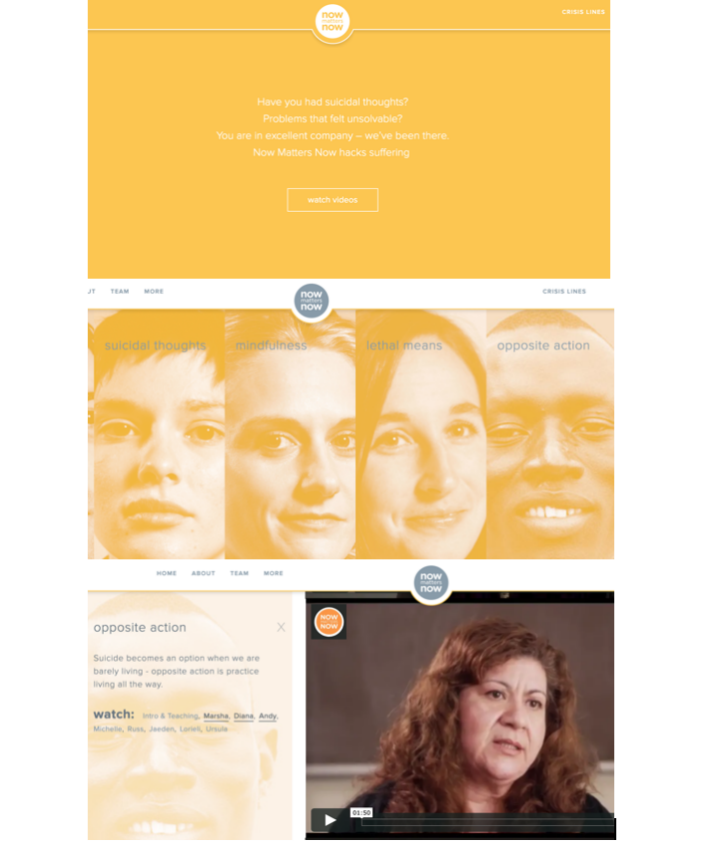
Website.

The website is designed to provide people experiencing suicidal thoughts and health care providers with DBT skills and to give health care providers a resource for supporting suicidal patients. The long-term goal is having providers use the skills in their own life and teach them to patients to help prevent and manage suicidal crises. As such, the website was developed with the provider audience in mind as a tool to help provide better care to their suicidal patients. The NowMattersNow.org landing page states “Have you had suicidal thoughts? Problems that felt unsolvable? You are in excellent company—we’ve been there.” This statement was intended to reduce stigma and signify that others having similar experiences were part of the website. Users proceed via a button to the home page. This page presents a panel of images of individuals with lived experience who have videos on the website. Images lead to topics such as DBT skills (opposite action, mindfulness, mindfulness of current emotion, paced breathing), suicidal thoughts (videos linking suicidal thoughts to difficulties managing emotions and DBT skills), and lethal means and caring messages (topics central to current care guidelines; Figure 1) [32]. Clicking on text in individual panels leads to videos on the topic with personal stories and didactic content. Home page menu options are About (text-based description of the website and a brief video provide an overview of the website), Team (bios and photos of Team Now Matters Now members allowing visitors more context about those featured in the videos), and More (eg, additional engagement options such as social media links, email sign-up, and downloadable “caring message” cards that providers can share with patients to direct them to the site). The home page also has a Crisis Lines option leading to national and international phone, short message service text message, and instant messaging options (also part of care guidelines). NowMattersNow.org is built on WordPress and instrumented with Google Analytics, which collects client-side statistics for how users find and interact with the site.

### User Experience Survey

**Figure 2:**
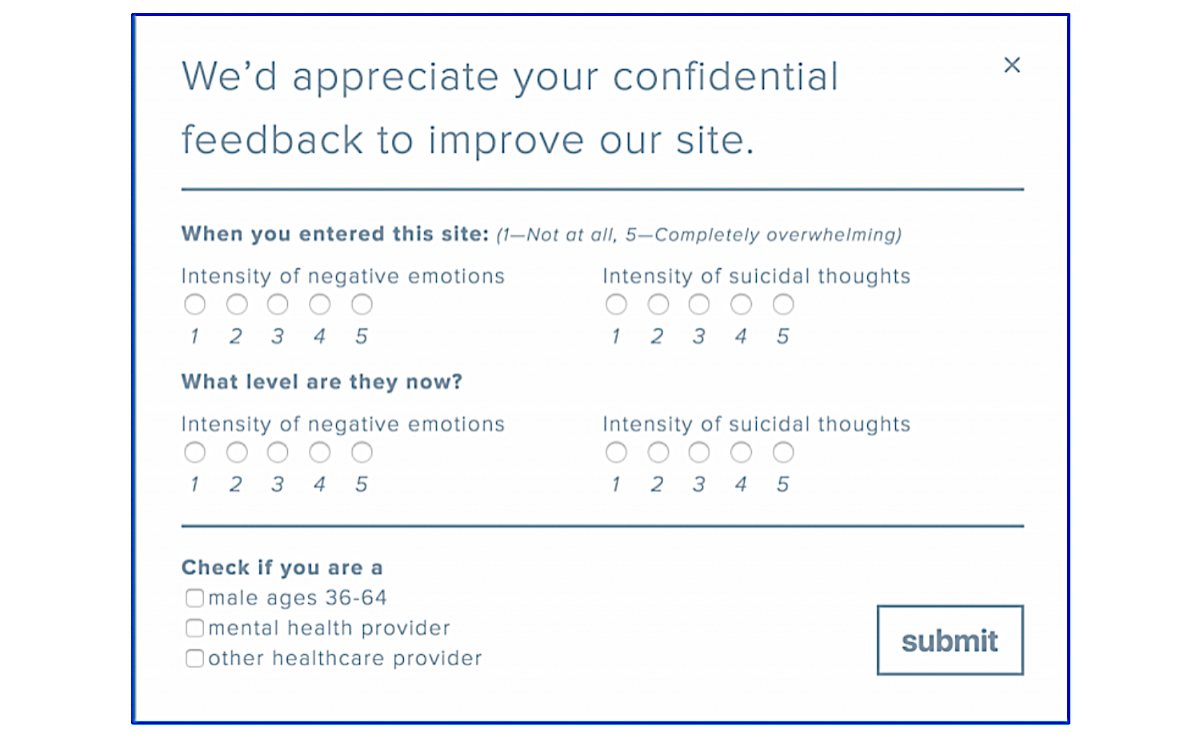
Survey.

NowMattersNow.org regularly collects survey data during a user’s session for quality improvement purposes and to determine to what degree certain populations use the website and if it helps them. The 7-item survey was built in WordPress using HTML elements triggered by JavaScript, is not advertised, and open (no log-in required). The header is, “We’d appreciate your confidential feedback to improve our site.” The survey asks users to retrospectively rate their “Intensity of negative emotions” and “Intensity of suicidal thoughts” as 1 to 5 (“Not at all” to “Completely Overwhelming;” Figure 2) for “When you entered this site” (baseline) and “What level are they now?” (post website use). Only 1 number can be selected for each question. These survey items were adapted from DBT skills “diary cards” used by patients to track their suicidal thoughts, mood, and related behaviors [20-22]. The survey also asks about demographics as nonexclusive checkboxes (ie, male aged 36-64 years, mental health provider, and health care provider). Survey responses were not assessed for completeness nor required for survey submission (although only complete surveys were used in analyses), and once submitted (using a “submit” button), all responses were final. Data that were entered, but not submitted, were not recorded. The methods of survey development were similar to best practices recommended by the US Department of Health and Human Services for developing Web-based surveys such as simplicity, clarity, and user centeredness [33]. The survey asks about suicidal thoughts and negative emotions, which likely correspond [34].

The survey displays after 1 min on any page viewed on mobile phones (timing determined by the survey software). On desktops and tablets, it originally displayed after 3 min (the approximate length of 1 short video) on a page and changed to 8 min (essentially the length of 2 short videos or 1 longer video) on August 7, 2015 to allow for longer time on the website before surveying users. We know that participants visited a page for the minimum time to receive the survey, but they could have visited several pages before triggering the survey. Without using cookies, we could track only how long users were on a page, not the website, and we are reluctant to use tracking features because of the sensitive website content. Once the survey surfaces, users cannot proceed until they fill out the survey, close its window using a button in the right-hand corner, or choose another option on the navigation bar. Once displayed (whether completed or not), the survey does not surface for that user for at least 24 hours. We do not know the number of times the survey surfaced and was not completed. The survey was tested by Civilization website developers before release and before data collection for this study started. We had no contact with the responders other than their survey submission.

### Study Sample and Study Data

This study analyzed a convenience sample of data from surveys completed from March 5, 2015, the first full day the survey was posted, to December 3, 2017, when data were retrieved. The personal information of Internet Protocol (IP) address snippets was removed from the dataset after assigning each participant a unique identification number. The final dataset was stored in Google Drive, to which authors UW, RN, AJW, XZ, DH had access. We did not track how long survey completers stayed on the website beyond the survey. The Allendale Institutional Review Board (Old Lyme, Connecticut) approved this study.

### Statistical Analyses

#### Primary Outcome

Longitudinal regression analyses used generalized estimating equations (GEEs; [35]) to evaluate the magnitude and statistical significance of user-reported changes in (1) suicidal ideation and (2) negative emotion. An exchangeable working correlation matrix in conjunction with cluster robust standard errors was estimated to account for correlation of repeated measures (baseline and post) within participants. A total of 2 sets of analyses were performed evaluating outcomes for both suicidal thoughts and negative emotions as (1) a continuous measure and (2) a dichotomous indicator of *high* (4 or 5) versus *low* ratings (3 or less). Logistic and Gaussian GEEs were used for binary and continuous variables, respectively. In the primary analyses evaluating changes in outcomes across all participants, each outcome variable was regressed on time point (post vs baseline).

#### Men Aged 36 to 64 Years and Health Care Providers

To evaluate if changes in outcomes differed by these demographic groups, primary analyses were extended to include indicator variables for (1) middle-aged male (=1), (2) mental health provider (=1), (3) other health provider (=1), and (4) the interaction of each demographic variable with time (post vs baseline). In stratified analyses, each outcome variable was regressed on middle-aged male, mental health provider, other health provider, middle-aged male by time (post vs baseline), mental health provider by time, and other health provider by time. The statistical test of the subgroup effects was the magnitude and statistical significance of each demographic variable by time interaction.

#### Mobile Versus Other Device and Delay to Survey

To evaluate if outcomes differed by electronic device type or delay before being presented with the survey, primary analyses were extended to include indicator variables for (1) device type (nonmobile vs mobile) and (2) survey delay interval (1, 3, 8 min). For device-type analysis, outcome variables were regressed on device type, time (post vs baseline), and the device type by time interaction. In delay-interval analysis, outcome variables were regressed on delay interval, time, and the delay interval by time interaction. Delay interval was divided into 2 planned contrasts of (1) 8 versus 1 min and (2) 3 versus 1 min. Statistical tests of device-type effects and delay-related effects were magnitude and statistical significance of (1) device type by time and (2) delay interval by time interactions.

## Results

### Website Visitors

During the time in which surveys were analyzed, 167,878 sessions (website visits) and 138,386 unique visitors were recorded. Of those sessions, 52% came to the website via “organic search terms,” 21% came directly to the website (bookmark or typing in the URL), 9% came via a link from social media, 9% came via paid advertisements for the site (Google AdWords), and 9% came via referrals (link from another website). On average, users viewed 2.1 pages per session and stayed on the website for 1 min 31 seconds. The average bounce rate (leaving after viewing 1 page) was 61%, and 18% of sessions were from returning visitors. People arrived via organic search terms when they searched for something that led to NowMattersNow.org being listed, and then clicked on our link, and the link was not a paid advertisement. To better understand how people found the website, Google search term data collection was enabled from December 11, 2017 to February 3, 2018 (54 days), which provided results that indicated that a number of organic search terms related to suicide and suicide help (eg, “crisis chat,” “crisis text line uk,” “suicide chat,” and “now matters now,” each individually making up no more that 4% of searches) represented the search terms leading to NowMattersNow.org. The Google Adwords were phrases we thought people who were suicidal might Google (“I want to die” and “how do I kill myself?”). If someone Googled such a term, they might see the website as an advertisement option. Geographically, visitors were largely from the United States (61%), Canada (15%), United Kingdom (9%), and Russia (3%), with Australia, Brazil, New Zealand, India, France, and Germany each making up 2% or less.

### Survey Responders

We received 3931 complete responses. Of these, 261 were removed because they were likely from people who previously completed the survey, identified from duplicate IP address snippets (keeping only earliest dated record). Surveys with incomplete data were not included in the analyses as the amount of missing data was minimal (6.7%) [36]. Of the 3670 unique survey responses, 514 (14% of survey sample) identified as men aged 36 to 64 years. Of survey completers, 460 (13%) identified as mental health professionals and 308 (8%) as other health care professionals, with 40 (1%) identifying as both. Users completed surveys after 1-min (2096; 57%), 3-min (388; 11%), or 8-min (1186; 32%) delays. Mobile users (57%) all received the survey at 1 min, therefore nonmobile device users comprised the remaining 43% (1574) of the sample (Table 1).

Survey ratings for suicidal thoughts pooled at the low and high ends of the scale, clustering toward low (1, not at all) and high (4 and 5, completely overwhelming). For baseline, the greatest number of responses were a score of 5 (28%), followed by 1 (28%), 4 (21%), 3 (17%), and 2 (7%). Post scores were 1 (32%), 2 (29%), 5 (23%), 4 (19%), and 3 (17%). In our sample, of those who reported suicidal thoughts (2644) at baseline, 29% (n=763) reported a 1 or more point reduction in suicidal thoughts pre versus post visit, the vast majority stayed the same (63%, n=1666), and 8% (n=215) rated suicidal thoughts 1 or more points worse (8%). Of those who reported worse scores, 2% (n=51) were more than a point worse, 1% (n=21) were 3 or 4 points worse.

### Primary Outcome Analyses

**Figure 3:**
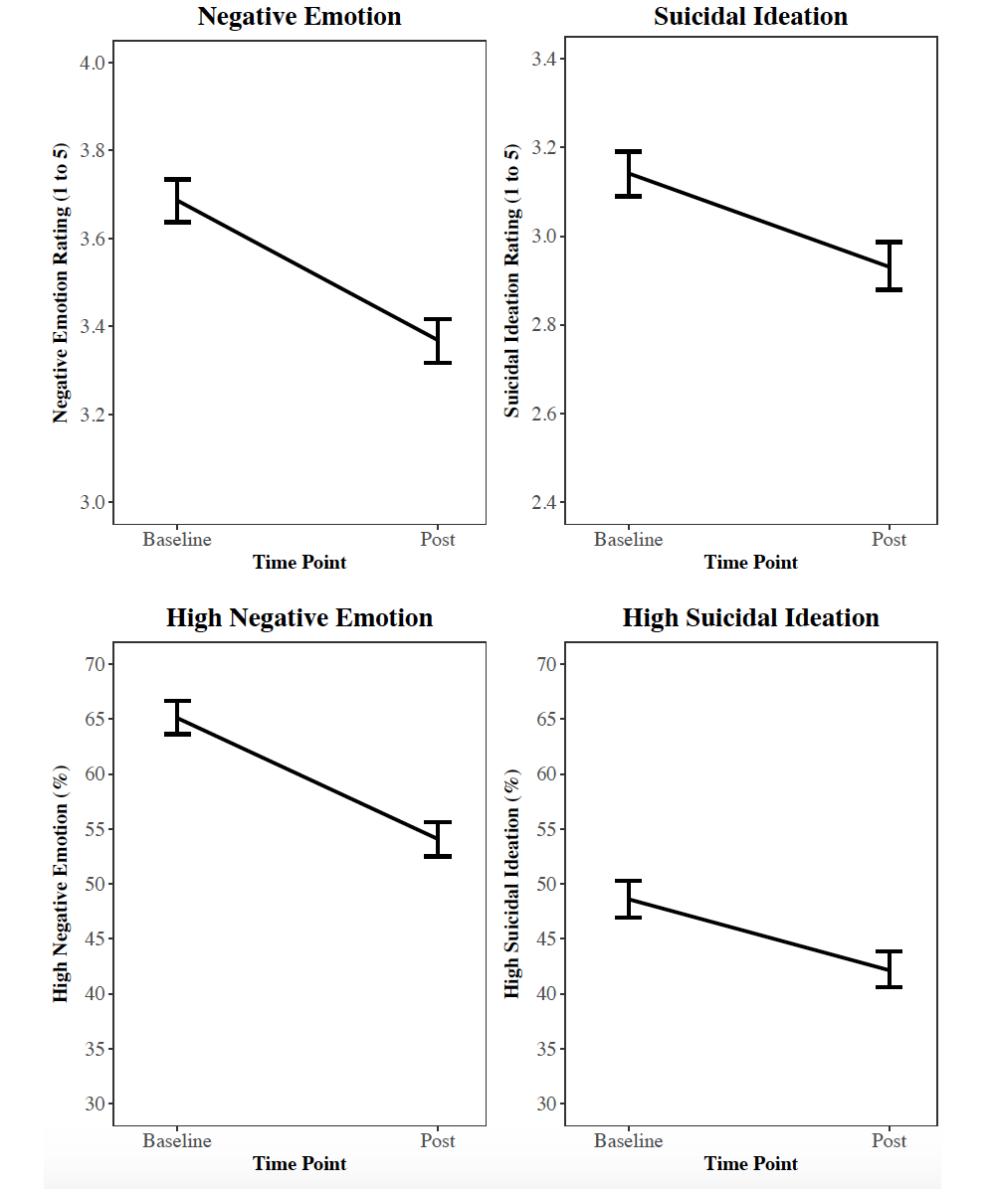
Change in negative emotion and suicidal ideation (continuous and dichotomous) from baseline to post website for all participants. Error bars indicate SDs.

The overall changes reported for all participants for the 4 main study outcomes (continuous and dichotomous for baseline vs post) is shown in Figure 3. Survey participants reported an average 0.21-point reduction (95% CI –0.24 to –0.19; *P*<.001; *d*=0.13) in suicidal ideation and a 0.32-point reduction (95% CI –0.35 to –0.29; *P*<.001; *d*=0.21) in negative emotion ratings (both 1 to 5 scale) after viewing the website. The average visitor’s probability of endorsing high suicidal ideation (4 or 5) decreased 6.4 percentage points (95% CI –7.55 to –5.33; *P*<.001; *d*=0.13) after viewing the website. The average visitor’s probability of endorsing high negative emotion (4 or 5 at baseline) decreased by 10.9 percentage points (95% CI –12.20 to –9.64; *P*<.001; *d*=0.23) after viewing the website.

**Table 1:** Demographic groups, electronic device type, and delay to survey (N=3670).

Outcome variable		Total	Men aged 36 to 64 years (n=512)	Mental health provider (n=460)	Other health care provider (n=308)	Mobile (all 1 min to survey) (n=2096)	8 min to survey (n=1186)	3 min to survey (n=388)
**Baseline suicidal thoughts**								
	Intensity, mean (SD)	3.1 (1.6)^a^	3.3 (1.6)^a^	1.8 (1.5)	2.8 (1.7)	3.6 (1.4)^a^	2.7 (1.6)^a^	2.2 (1.5)^a^
	Percentage low (score=1), n (%)	1012 (28)	131 (26)	331 (72)	130 (42)	315 (15)	486 (41)	215 (55)
	Percentage high (score=4-5), n (%)	1784 (49)^a^	277 (54)^a^	86 (19)	122 (40)	1235 (59)^a^	448 (38)^a^	101 (26)^a^
**Baseline negative thoughts**								
	Intensity, mean (SD)	3.7 (1.5)^a^	3.7 (1.5)^a^	2.2 (1.6)^a^	3.1 (1.7)^a^	4.1 (1.3)^a^	3.3 (1.6)^a^	2.8 (1.6)^a^

^a^Indicates significant reduction from baseline to post *P*<.001, not applicable from baseline suicide ideation.

#### Men Aged 36 to 64 Years

**Figure 4:**
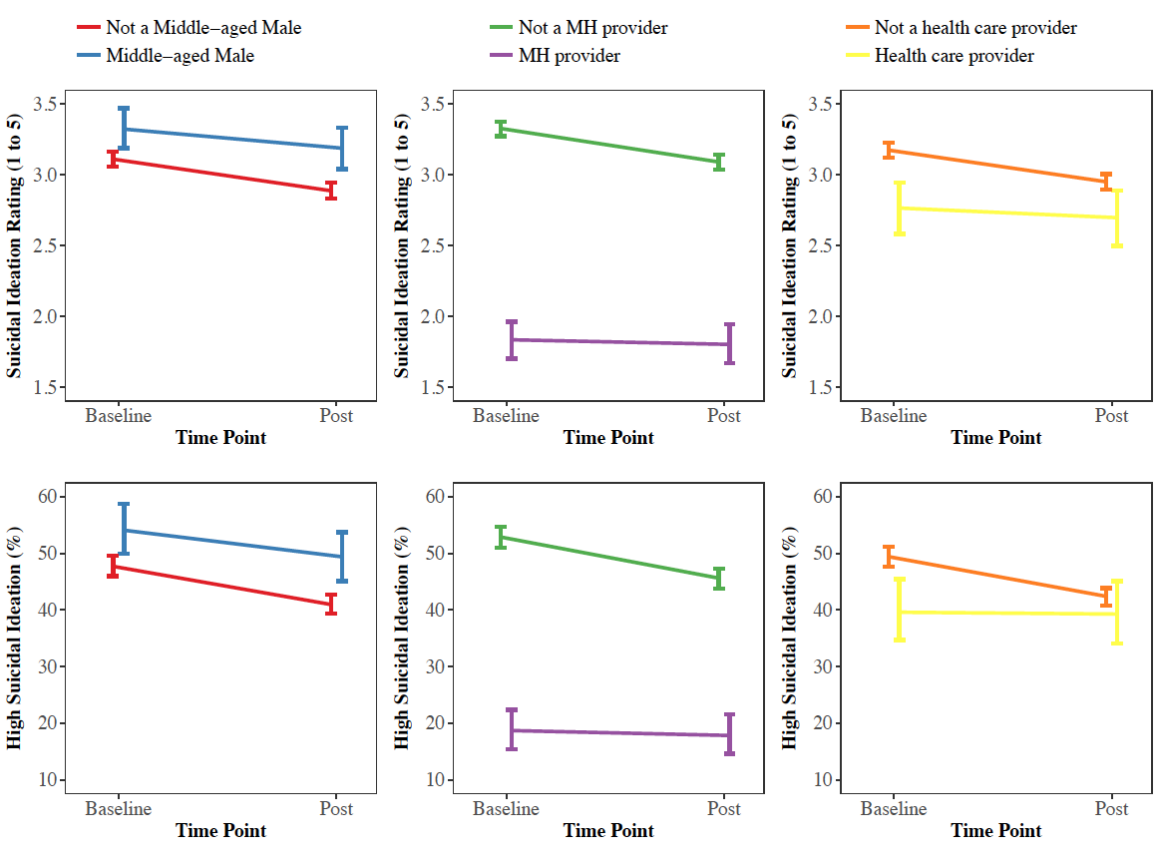
Average suicidal ideation and high suicidal ideation ratings from baseline to post website by demographic group. MH: mental health.

Men aged 36 to 64 years reported their overall severity of suicidal ideation decreased on the 1 to 5 scale (Estimate, est=–0.13; 95% CI –0.19 to –0.07; *P*<.001; *d*=–0.08), although the decrease was 0.09 points greater (*d*=0.06; *P*<.001) among nonmiddle-aged males (est=-0.22; 95% CI –0.25 to -0.20; *P*<.001; *d*=0.14). High suicidal ideation decreased for middle-aged males (%=–4.74; 95% CI –7.38 to –2.10; *P*<.001; *d*=0.09). Results were similar and significant for overall negative emotions (est=–0.21; 95% CI –0.29 to –0.14; *P*<.001; *d*=0.14) and for the probability of high negative emotion (%=–6.90; 95% CI –10.70 to –3.11; *P*<.001; *d*=0.14; Figure 4).

#### Health Care Providers

For mental health providers, there were no changes observed for overall severity of suicidal ideation from baseline to post (est=–0.03; 95% CI –0.07 to 0.01; *P*=.10; *d*=0.02) or for the probability of endorsing high suicidality (%=–1.36; 95% CI –2.86 to 0.14; *P*=.08; *d*=0.03). This finding was consistent with other health care providers for suicide ideation overall (est=–0.07; 95% CI –0.15 to 0.02; *P*=.12; *d*=0.04) and high suicidal ideation (%=–0.54; 95% CI –3.95 to 2.87; *P*=.76; *d*=0.01). However, significant improvements were seen in negative emotions overall for mental health providers (est=–0.18; 95% CI –0.24 to –0.12; *P*<.001; *d*=0.12) and high negative emotions (%=–4.17; 95% CI –6.39 to –1.95; *P*<.001; *d*=0.09) as well as other health care providers (Overall: est=–0.18; 95% CI –0.29 to –0.07; *P*=.001; *d*=0.12 and high rating: %=–5.59; 95% CI –10.04 to –1.14; *P*=.01; *d*=0.12; Figure 4).

#### Mobile Versus Other Device and Delay to Survey

For website platform (mobile vs other), suicidal ideation severity decreased for both desktop or tablet users (est=–0.21; 95% CI –0.25 to –0.17; *P*<.001; *d*=0.13) and mobile users (est=–0.21; 95% CI –0.25 to –0.18; *P*<.001; *d*=0.13). High suicidal ideation decreased for both desktop or tablet users (%=–6.99; 95% CI –8.59 to –5.38; *P*<.001; *d*=0.14) and mobile users (%=–6.06; 95% CI –7.59 to –4.52; *P*<.001; *d*=0.12). Similarly, negative emotion severity decreased for both desktop or tablet users (est=–0.34; 95% CI –0.38 to –0.29; *P*<.001; *d*=0.23) and mobile users (est=–0.30; 95% CI –0.34 to –0.26; *P*<.001; *d*=0.20). In addition, high negative emotions decreased for both desktop or tablet users (%=–10.67; 95% CI –12.55 to –8.79; *P*<.001; *d*=0.22) and mobile users (percentage=–11.26; 95% CI –13.07 to –9.45; *P*<.001; *d*=0.24).

For survey delay interval (1, 3, 8 min), suicidal ideation severity decreased for those surveyed after delays of 1 min (est=–0.21; 95% CI –0.25 to –0.18; *P*<.001; *d*=0.13), 3 min (est=–0.18; 95% CI –0.24 to –0.12; *P*<.001; *d*=0.11), and 8 min (est=–0.22; 95% CI –0.26 to –0.18; *P*<.001; *d*=0.14). High suicidal ideation also decreased for those surveyed after delays of 1 min (%=–6.06; 95% CI –7.59 to –4.52; *P*<.001; *d*=0.12), 3 min (%=–5.67; 95% CI –8.38 to –2.96; *P*<.001; *d*=0.11), and 8 min (%=–7.42; 95% CI –9.36 to –5.48; *P*<.001; *d*=0.15). Similarly, negative emotion severity decreased for surveyed after delays of 1 min (est=–0.30; 95% CI –0.34 to –0.26; *P*<.001; *d*=0.20), 3 min (est=–0.29; 95% CI –0.37 to –0.21; *P*<.001; *d*=0.19), and 8 min (est=–0.35; 95% CI –0.40 to –0.30; *P*<.001; *d*=0.24). High negative emotions also decreased for those surveyed after delays of 1 min (%=–11.26; 95% CI –13.07 to –9.45; *P*<.001; *d*=0.24), 3 min (%=–10.05; 95% CI –13.37 to –6.74; *P*<.001; *d*=0.21), and 8 min (%=–10.88; 95% CI –13.12 to –8.63; *P*<.001; *d*=0.23).

#### Sensitivity Analyses Correcting for Multiple Statistical Tests

All findings (*P*<.05) remained statistically significant after accounting for multiple testing using the Benjamini-Hochberg procedure [37].

## Discussion

### Principal Findings

The results of our evaluation of NowMattersNow.org suggest that this Web-based resource has the potential to address several major challenges to caring for suicidal patients. First, NowMattersNow.org was developed to address a historic need for suicidal patients [15] by providing tailored content to support suicidal individuals and the providers who care for them [19]. During our 32-month evaluation period, there were 138,386 unique visitors to NowMattersNow.org, indicating this site was indeed receiving a fair amount of visitation. Next, over 70% of survey completers recalled having some degree of suicidal thoughts when they arrived at the website. Survey participants, overall, also reported significant reductions in suicidal thoughts. Highly suicidal respondents reported significant decreases in intensity of suicidal thoughts from website entrance to survey. Reductions in suicidal thoughts were matched by similar declines in reported intensity of negative emotions while visiting the website. This result indicated that the site attracted people who are experiencing suicidal thoughts, often at a high level of intensity. The results also suggest that website visits were associated with reductions in suicidal thoughts and negative emotions.

Finally, we examined different types of visitors and found that significant reductions in suicidal thoughts and negative emotions were largely consistent across subgroups. Middle-aged men reported significant reductions in suicidal thoughts and negative emotions. Given that this group makes up 38% of all suicides [35], these results are promising. However, reductions for middle-aged men were not as large as for the rest of the sample; therefore, the website could use greater tailoring toward men. For example, male identification on the survey could trigger an additional box requesting suggestions for improvements or their contact information for follow-up interview or survey about the same topic. About one-fifth of responders identified as mental health or other health care providers, which was overrepresented compared with the general population. These responders reported lower levels of suicidal thoughts and negative emotions. Health care providers likely have visited the website for reasons other than self-help, such as for providing a referral for a patient, as the website is part of clinician training efforts. These findings suggest that the website content is most useful for those for whom it is directly intended: those experiencing suicide ideation. Given the intentional simplicity of the survey, we could not glean information about the motivations for initial visits to the website (eg, experiencing severe negative emotions vs visiting for work). In the future, the survey could be developed to have an opt-out option for health care providers or those visiting the website for reasons not related to their own mental health, but this may also create unintentional stigma toward having suicidal thoughts. When we looked separately at user platform (mobile, desktop, and tablet), positive reductions were found in each group. We found this result to be promising because it may mean the website was showing benefit for youth, given that mobile users tend to be younger [38]. Indeed, the demographic category “age 12-18” was added to the user-experience survey in 2018, and results of these are forthcoming. Interestingly, mobile device users had higher severity ratings across both time points. Therefore, we may seek ways to target mobile audiences who, based on self-report, may be at greater risk for suicide. When we looked at time on website pages before the survey surfaced (1, 3, or 8 min), positive reductions were also consistent across times. This result meant that some users may find benefit in as little as 1 min on the website. We cannot separate the 1-min survey and mobile findings, but both were positive for reducing suicidal ideation.

### Limitations

There are important limitations to this study. The primary limitation is that user-experience data were collected at one point in time from individual users, without a control-group comparison. Distraction alone may have had an effect, or the passage of time may be associated with similar reductions. In addition, we do not know if experienced improvements lasted beyond the brief period that the survey covered. In addition, nonresponse bias may account for the findings—these findings may not generalize to people who saw the survey but did not complete it or people who never saw the survey who may not have had positive responses. It is possible that the nature of the survey topic (suicide) may have made some individuals less likely (eg, among those with greater self-stigma or experienced discrimination) or more likely (eg, experiencing the survey as highly relevant) to participate, including those potentially at higher risk of suicide. Another limitation is that participants were asked to rate their pre and post website suicidal thoughts and negative emotions on a single survey. Although this format was designed to maximize survey response, reported reductions in distress may be at least partially attributable to recall bias. Furthermore, the survey has face validity, but other types of validity have not been examined, and we do not know if survey completers represent typical suicidal patients seen in health care settings, our target audience. Finally, although changes in suicidal thoughts and emotions might occur through a number of mechanisms (eg, didactic information, exposure to evidence-based interventions, distraction from emotional suffering, referral to crisis services, return to baseline, placebo effect, and viewing videos of those with lived experiencing discussing stories of recovery), we could not evaluate these mediators given survey limits. Future randomized trials need to examine if this website (or which elements of this website), compared with other sites, is associated with similar or greater reductions in suicidal thought reductions. An in-progress randomized controlled trial comparing risk of suicide attempt (over 18 months) among patients reporting suicidal ideation will shed additional light on the utility of its content. Patients invited to a skills training intervention, utilizing a guided interactive version the NowMattersNow.org website, will be compared with patients randomized to usual care [39].

### Conclusions

NowMattersNow.org is a Web-based resource with the potential to help address gaps in suicide prevention and treatment resources for mental health professionals. Our findings supported use of the website as originally intended—as a support tool for providers who identify suicidal patients in their practice. In light of our results indicating website use is associated with reductions in suicidal thoughts, we suggest that health care providers can, with some confidence, refer suicidal people for short-term management of suicidal thoughts and negative emotions. For example, when suicidal patients are discovered as part of routine screening for depression, providers could share this website with the patient as part of safety planning procedures, which includes a list of options for managing suicidal thoughts [40]. Indeed, a number of health care providers are directed to the website through the Zero Suicide initiative, which has trained over 500 health systems to provide better suicide care. The resource is shared in nearly all of these trainings [41].

## Appendix

Multimedia Appendix 1 NowMattersNow.org introduction video.

## Abbreviations

DBT: dialectical behavior therapy
GEE: generalized estimating equation
IP: Internet Protocol
